# Kutane Infektionen mit nicht‐tuberkulösen Mykobakterien: eine retrospektive Studie mit 94 Fällen aus Deutschland

**DOI:** 10.1111/ddg.15910_g

**Published:** 2026-05-05

**Authors:** Luisa Bopp, Nicolai Deresz, Henning Klapproth, Isabelle Suárez, Jonathan Jantsch, Mario Fabri, Esther von Stebut

**Affiliations:** ^1^ Klinik für Dermatologie und Venerologie Medizinische Fakultät und Uniklinik Köln Universität zu Köln; ^2^ Abteilung für Dermatologie Universitätsklinik Johannes‐Gutenberg‐Universität, Mainz; ^3^ Innere Medizin I Medizinische Fakultät und Uniklinik Köln; ^4^ Institut für Medizinische Mikrobiologie Immunologie und Hygiene Medizinische Fakultät und Uniklinik Köln; ^5^ Zentrum für Molekulare Medizin Medizinische Fakultät Universität zu Köln; ^6^ Deutsches Zentrum für Infektionsforschung (DZIF) Partnerstandort Bonn‐Köln, Köln; ^7^ Klinik für Hautkrankheiten Universitätsklinikum Jena

**Keywords:** Aquarium‐Granulom, Mykobakterien, Mycobacterium marinum, NTM, fish tank granuloma, mycobacteriae, Mycobacterium marinum, NTM

## Abstract

**Hintergrund:**

Kutane nichttuberkulöse Mykobakterien‐Infektionen (NTM) sind diagnostisch und therapeutisch herausfordernd. Ziel dieser Studie war die Charakterisierung kutaner NTM‐Infektionen in Deutschland über einen Zeitraum von 24 Jahren.

**Patienten und Methodik:**

Eingeschlossen wurden 73 Patienten mit kutanen NTM‐Infektionen, die zwischen 2000 und 2011 an 17 verschiedenen deutschen Universitätskliniken diagnostiziert wurden, und 21 Patienten, die zwischen 2010 und 2024 in der Dermatologie der Universitätsklinik Köln behandelt wurden. Erhoben wurden demografische und klinische Daten sowie Informationen zu Diagnostik, Therapie und Therapieverlauf.

**Ergebnisse:**

Insgesamt wurden 94 Fälle analysiert (71% Männer, Durchschnittsalter 50 Jahre, > 75% immunkompetent). Eine Aquarium‐Exposition war der häufigste Risikofaktor. Der Erregernachweis mittels Nukleinsäureamplifikationstest und/oder Kultur gelang in 76% der Fälle. Der häufigste Erreger war *Mycobacterium (M.) marinum* (> 65%), gefolgt von *M. abscessus/chelonae*. Eine bestehende Immunsuppression war mit anderen NTM als *M. marinum* assoziiert. Über 90% der Patienten erhielten eine orale Antibiotika‐Therapie, in mehr als der Hälfte als Monotherapie (meist Clarithromycin). Die häufigste Kombination war Clarithromycin plus Rifampicin. Die mittlere Therapiedauer betrug mehr als 4 Monate. Komplikationen, unerwünschte Ereignisse und Rückfälle traten selten auf.

**Schlussfolgerungen:**

Unsere Ergebnisse unterstreichen die Notwendigkeit standardisierter Diagnoseverfahren und Therapieempfehlungen für kutane NTM‐Infektionen.

## EINLEITUNG

Nichttuberkulöse Mykobakterien (NTM) sind säurefeste Stäbchenbakterien, die ubiquitär in der Umwelt vorkommen, vor allem in natürlichen Gewässern, Böden und Trinkwasserverteilungssystemen.^1^ Nur einige von ihnen verursachen Erkrankungen beim Menschen und sind fakultativ pathogen. Die Manifestation einer Erkrankung hängt von der Interaktion des Mykobakteriums mit dem Immunsystem des Wirts ab. Immunsuppression, zum Beispiel bei Personen mit HIV oder nach Organtransplantation, sowie Biologika‐Therapien mit Tumornekrosefaktor (TNF)‐Rezeptorblockern erhöhen die Anfälligkeit und stellen einen Risikofaktor für disseminierte Infektionen dar.^2^, ^3^, ^4^ Pulmonale Erkrankungen sind die häufigste klinische Form der NTM‐Infektion, gefolgt von Haut‐ und Weichteilinfektionen.^5^

Kutane NTM‐Infektionen entstehen durch eine direkte Inokulation über kleine Hautverletzungen oder durch eine hämatogene Disseminierung einer systemischen Infektion.^6^ Sie können in zwei Gruppen unterteilt werden: lokale oberflächliche Infektionen (einschließlich Weichgewebeinfektionen, Abszesse, Knoten, Pusteln) und tiefe Infektionen (subfasziale Abszesse, Tenosynovitis).^7^ Die Inkubationszeit reicht von 2 bis hin zu 6 Wochen.^8^ Auch wenn das histopathologische Spektrum von NTM‐Infektionen variabel ist, ist die Granulombildung ein häufiges Muster.^9^, ^10^ Obwohl die tatsächliche Häufigkeit von kutanen NTM‐Infektionen nicht bekannt ist, wurde in den letzten Jahren ein Anstieg der Inzidenz vermutet, der vor allem auf vermehrte immunsuppressive Therapien, chirurgische/kosmetische Verfahren und eine verbesserte Diagnostik zurückzuführen ist.^11^, ^12^, ^13^, ^14^ Frühere Traumata (zum Beispiel Liposuktion) sind mit Infektionen durch schnell wachsende Mykobakterien (*rapidly growing mycobacteria* [RGM] wie *Mycobacterium [M.] fortuitum*, *M. abscessus* und *M. chelonae*), vor allem über kontaminierte Wasserversorgungssysteme, assoziiert.^15^, ^16^, ^17^ Zusammen mit *M. marinum*, dem Haupterreger bei Aquariumsbesitzern und anderen wasserexponierten Personen,^3^, ^18^ sind sie die am häufigsten isolierten Erreger bei kutanen NTM‐Infektionen. Diagnose und Behandlungsbeginn verzögern sich häufig aufgrund unspezifischer Klinik und fehlendem Verdacht. Weiterhin ist die Diagnostik von NTM‐Infektionen komplex,^19^ zeitaufwändig und der Erregernachweis nicht immer erfolgreich.

Zur Therapie von NTM‐Infektionen der Haut gibt es keine evidenzbasierten Empfehlungen. Da randomisierte, kontrollierte Studien fehlen, beruhen Behandlungsempfehlungen auf Expertenmeinungen.^20^ Darüber hinaus sind die gemeinsamen Behandlungsansätze an die jüngste Leitlinie aus dem Jahr 2007 angepasst, die sich hauptsächlich auf pulmonale Infektionen bezieht.^20^ Im Allgemeinen wird eine Mono‐ oder duale Antibiotikatherapie über mehrere Monate, manchmal in Kombination mit chirurgischen Maßnahmen, empfohlen.

In dieser retrospektiven Studie untersuchten wir die klinischen und mikrobiellen Merkmale von kutanen NTM‐Infektionen in Deutschland über einen Zeitraum von 24 Jahren. Eingeschlossen wurden 73 Patienten, die zwischen 2000 und 2011 in 17 verschiedenen deutschen Universitätskliniken diagnostiziert wurden (Kohorte 1)^21^, und 21 Patienten, die in unserer Klinik in Köln zwischen 2010 und 2024 behandelt wurden (Kohorte 2). Zusätzlich zu demographischen und klinischen Daten umfasst unsere Analyse auch Daten zu verschiedenen diagnostischen Verfahren und Therapien. Darüber wurden die histopathologischen Präparate der Kohorte‐2 erneut auf Hautreaktionsmuster reevaluiert.

## PATIENTEN UND METHODIK

In Kohorte 1 wurden die Akten von Patienten mit kutanen NTM‐Infektionen ausgewertet, die sich zwischen 2000 und 2011 in 17 verschiedenen universitären Hautkliniken in Deutschland vorstellten.^21^ In Kohorte 2 wurden die Daten aus Krankenakten von Patienten analysiert, die zwischen dem 01.01.2010 und 31.03.2024 an der Klinik und Poliklinik für Dermatologie der Universitätsklinik Köln behandelt wurden. Tiefe Infektionen wurden nicht identifiziert. Neben demographischen Daten, prädisponierenden Faktoren, Reiseanamnese und der Symptomdauer wurden die klinische Präsentation, diagnostische und therapeutische Strategien sowie das Outcome erfasst.

Es wurde analysiert, auf welchen diagnostischen Befunden die abschließende Diagnose basierte (direkter NTM‐Nachweis und histopathologischer Befund von Hautbiopsien, kutane Manifestationen, Risikofaktoren wie Kontakt zu Aquarien, und das Ansprechen auf eine antimykobakterielle Therapie). Ein positiver Erregernachweis wurde definiert als der Nachweis von NTM mittels Nukleinsäureamplifikationstest (*nucleic acid amplification test*, NAAT) und/oder kultureller Anzucht aus einem Infektionsherd. Die Zeit bis zur Diagnose wurde als Zeitraum zwischen Symptombeginn und Diagnosestellung definiert. Ein Rezidiv lag vor, wenn sich beim Abschlussbesuch nach Beendigung der Therapie erneut klinische Zeichen der Erkrankung zeigten. Fälle ohne dokumentierten Verlauf oder ohne Abschlussuntersuchung wurden als *lost to follow‐up* gewertet. Die histopathologischen Präparate der Fälle aus Kohorte 2 wurden erneut detailliert begutachtet, um epidermale Veränderungen, den Typ der granulomatösen Reaktion (suppuratives Granulom, nicht‐verkäsender tuberkuloider Typ, *messy granuloma*) sowie Zusammensetzung, Tiefe und Verteilung des (begleitenden) Immuninfiltrats zu erfassen.^22^, ^23^, ^24^

Daten aus Kohorte 1 und Kohorte 2 wurden getrennt und – sofern möglich – gemeinsam analysiert. Qualitative Variablen wurden als absolute und relative Häufigkeiten dargestellt, quantitative Variablen als Mittelwert (Bereich) beziehungsweise Median (Interquartilsabstand, IQR). Chi‐Quadrat‐Tests wurden mithilfe der Software Prism Version 10.1.2 (GraphPad) durchgeführt.

## ERGEBNISSE

Die Patientencharakteristika beider Kohorten sind in Tabelle 1 dargestellt. Insgesamt wurden 94 Fälle identifiziert (n = 73 in Kohorte 1, n = 21 in Kohorte 2). Der Großteil der Patienten war männlich (74% in Kohorte 1, 69% in Kohorte 2). Das durchschnittliche Alter betrug 50 Jahre (Bereich: 2–83). In der Mehrzahl der Fälle (jeweils 85% in beiden Kohorten) bestand kein Zusammenhang mit Auslandsreisen. Der häufigste Risikofaktor war der Kontakt mit Aquarien (79,5% in Kohorte 1, 33,5% in Kohorte 2); in beiden Kohorten zusammen wurden fünf berufsbedingte Expositionen dokumentiert. In Kohorte 1 traten signifikant mehr Fälle mit Wasserexposition auf, insbesondere mit Aquarien, während in Kohorte 2 häufiger keine spezifische Exposition dokumentiert war (p = 0,0009).

**TABELLE 1:** Patientencharakteristika.

			Deutschland (2000–2011)	Köln (2012–2024)	Kombiniert (2000–2024)
*Patientenanzahl*	*Alle*		*73*	*21*	*94*
Biologisches Geschlecht – n (%)	Weiblich Männlich Unbekannt		18 (24,7) 54 (74) 1 (1,3)	8 (38,1) 13 (61,9) 0 (0)	26 (27,7) 67 (71,3) 1 (1,1)
Alter bei Diagnose	Mean (Bereich) – J Median (IQR) – J Unbekannt – n (%)		50,5 (2–82) 51 (39,5–64,5) 10 (13,7)	48,3 (15–83) 51 (32–58) 0 (0)	50 (2–83) 51 (39–63) 10 (10,1)
Reiseanamnese – n (%)	Ja Nein Unbekannt		3 (4,1) 62 (84,9) 8 (11)	3 (14,3) 18 (85,7) 0 (0)	6 (6,4) 80 (85,1) 8 (8,5)
Exposition – n (%)	Aquarium Pool/Teich Vorangegangene Liposuktion Keine spezifische Exposition		58 (79,5) 4 (5,5) 0 (0) 11 (15,1)	7 (33,3) 3 (14,3) 1 (4,8) 10 (47,6)	65 (69,1) 7 (7,4) 1 (1,1) 21 (22,3)
Berufsbedingte Infektion – n (%)	Ja Nein Unbekannt		3 (4,1) 55 (75,3) 15 (20,6)	2 (9,5) 18 (85,7) 1 (4,8)	5 (5,3) 73 (77,7) 16 (17)
Lokalisation der Infektion – n (%)	Obere Extremität Untere Extremität Stamm/Hals Gesicht Unbekannt		65 (89) 4 (5,5) 0 (0) 3 (4,1) 1 (1,4)	13 (61,9) 5 (23,8) 2 (9,5) 1 (4,8) 0 (0)	78 (83) 9 (9,6) 2 (2,1) 4 (4,3) 1 (1,1)
Immunosuppression – n (%)	Ja Nein	Orales Steroid HIV Methotrexat Ciclosporin Rituximab, CLL TNF‐Inhibitor Azathioprin	7 (9,6) 3 (42,9) 2 (28,6) 1 (14,25) 1 (14,25) 0 (0) 0 (0) 0 (0) 66 (90,4)	5 (23,8) 0 (0) 0 (0) 0 (0) 0 (0) 1 (4,8) 3 (14,3) 1 (4,8) 16 (76,2)	12 (12,8) 3 (3,2) 2 (2,1) 1 (1,1) 1 (1,1) 1 (1,1) 4 (4,3) 1 (1,1) 82 (87,2)
Symptomdauer bis zur ersten Vorstellung im Krankenhaus (Tage)	Mean (Bereich) Median (IQR)		122 7–1095 40 28–89	258,9 42–1095 150 80–312	182 7–1095 80 35–211

*Abk*.: CLL, chronisch‐lymphatische Leukämie; HIV, humanes Immundefizienz‐Virus; IQR, Interquartilsabstand; TNF, Tumornekrosefaktor; J, Jahr

**ABBILDUNG 1:**
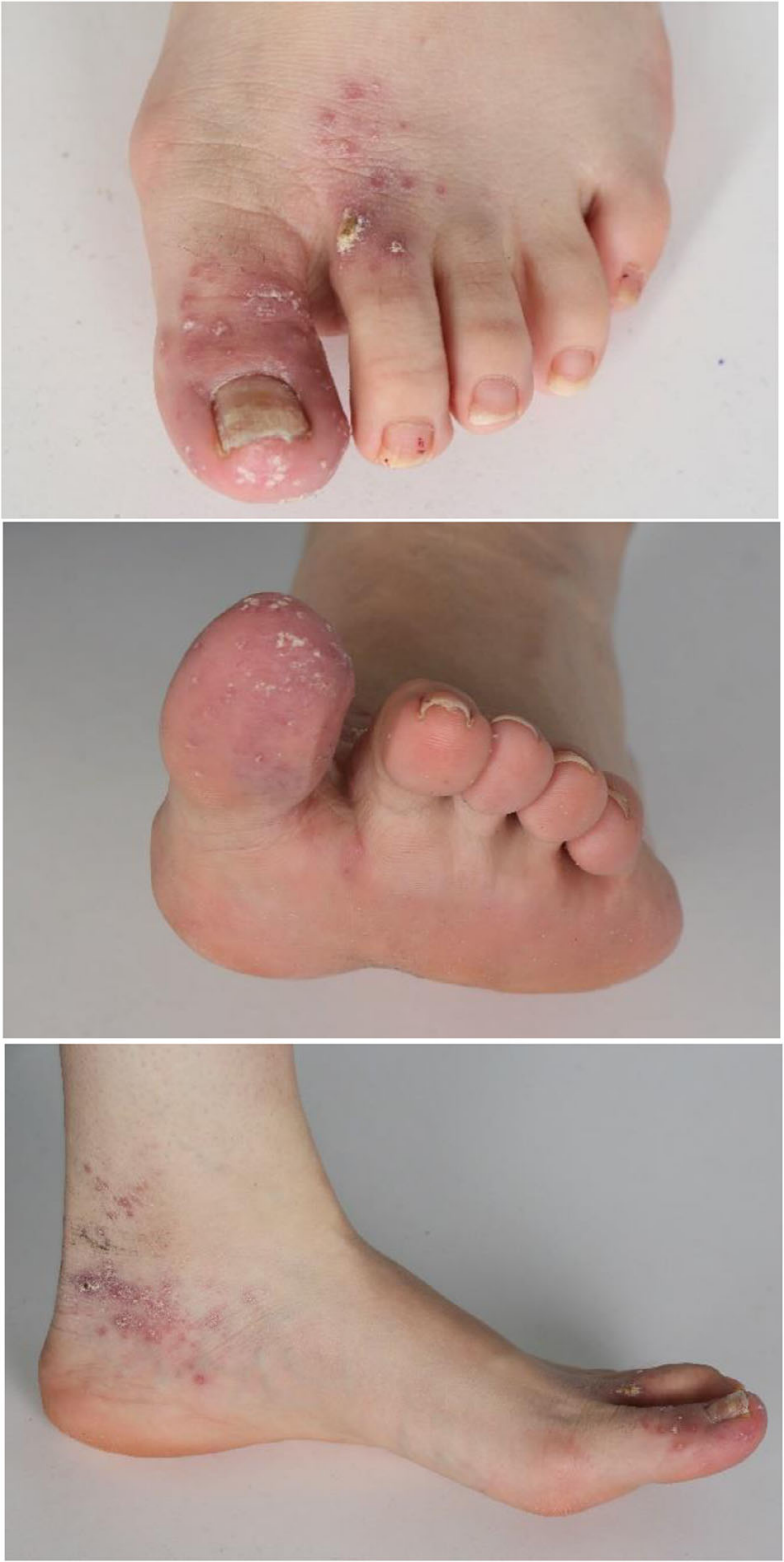
*Mycobacterium‐marinum*‐Infektion des linken Fußes und der Zehen bei einer Patientin mit Morbus Crohn unter Therapie mit Adalimumab und Mesalazin. Multiple gruppierte granulomatöse Papeln und Plaques mit Krusten an der Großzehe, dem dorsalen Vorfuß sowie am medialen Sprunggelenk.

**ABBILDUNG 2:**
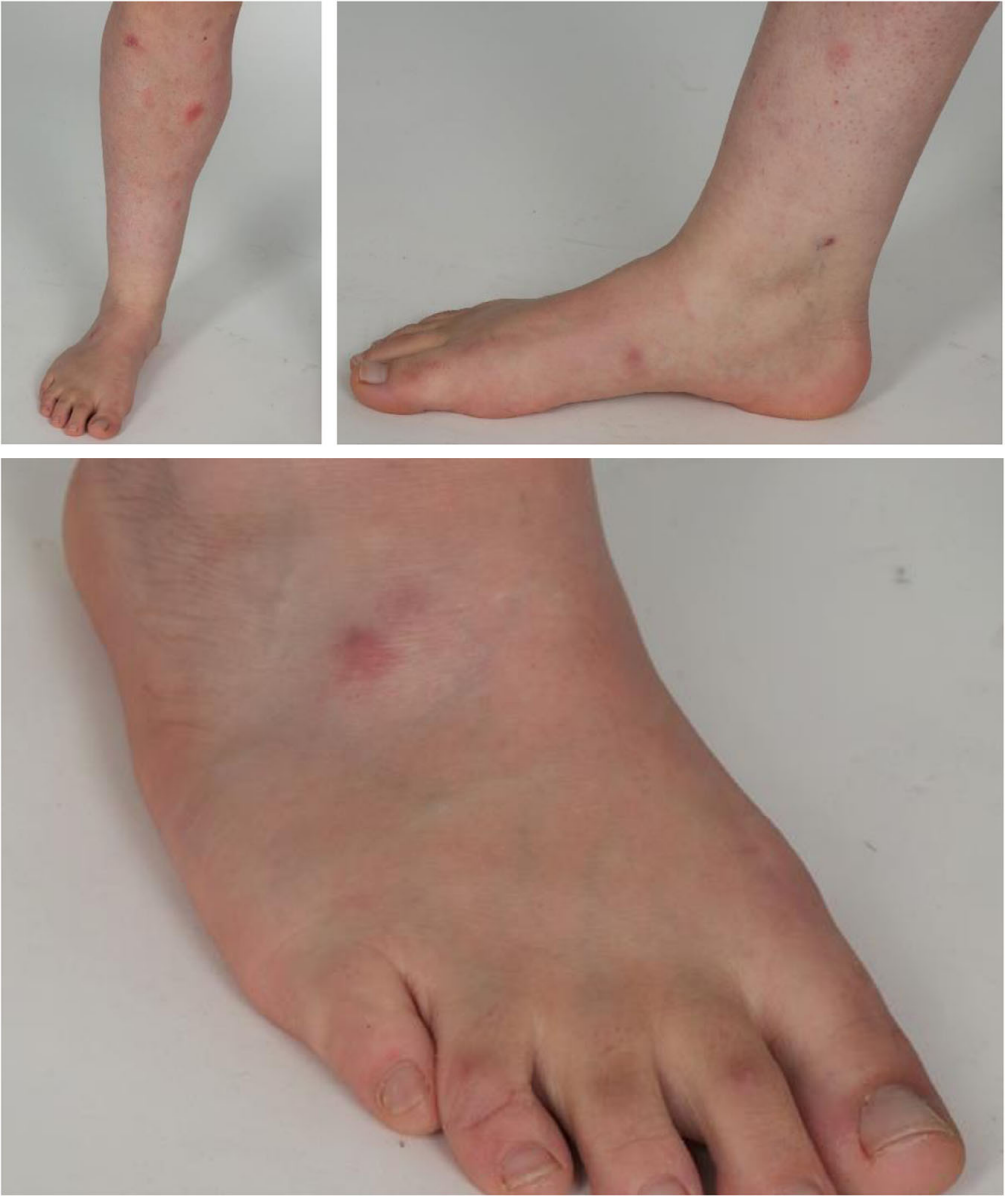
Kutane Infektion mit *M. abscessus complex* mehrere Wochen nach Liposuktion bei einer Patientin unter Immunsuppression mit Azathioprin. Multiple subkutane erythematöse Knoten und Plaques des rechten Fußes und Unterschenkels.

Die häufigste Lokalisation war die obere Extremität (89% in Kohorte 1, 62% in Kohorte 2) (Tabelle 1). Die Hautveränderungen präsentierten sich typischerweise als solitäre oder wenige erythematöse Papeln beziehungsweise Knoten (Abbildungen 1, 2), häufig mit Erosionen oder Ulzerationen beziehungsweise Hyperkeratosen, teils begleitet von angrenzenden Erythemen und in sporotrichoider Verteilung. Der Großteil der Patienten war immunkompetent (90% in Kohorte 1, 76% in Kohorte 2). Eine Therapie mit TNF‐Rezeptor‐Blockern bestand in 4/21 Fällen der Kohorte 2 (19%). Die mittlere Symptomdauer bis zur Erstvorstellung in der Klinik betrug in Kohorte 1 122 Tage (Bereich: 7–1095) und in Kohorte 2 259 Tage (Bereich: 42–1095) (Tabelle 1). Vor der Vorstellung an einer Universitätsklinik waren 15/73 Fälle (21%) aus Kohorte 1 und 7/21 Fälle (33%) aus Kohorte 2 zunächst von Hausärzten oder niedergelassenen Dermatologen fehldiagnostiziert worden und hatten eine empirische antibiotische Therapie gegen Streptokokken oder Staphylokokken erhalten.

Während in allen Fällen der Kohorte 2 eine standardisierte Diagnostik mittels Hautbiopsien für Histologie, NAAT und mikrobiologische Kultur erfolgte, waren die diagnostischen Vorgehensweisen in Kohorte 1 heterogener (Tabelle 2): In 47/73 Fällen (64,4%) wurde eine Kombination aus Histologie und NAAT und/oder Kultur durchgeführt, während in 14/73 Fällen (19%) ausschließlich ein direkter Erregernachweis mittels NAAT und/oder Kultur ohne histologische Untersuchung erfolgte. In zehn Fällen der Kohorte 1 erfolgte ausschließlich eine histopathologische Untersuchung, und in zwei Fällen wurde die Diagnose allein klinisch gestellt. Ein direkter Erregernachweis mittels Nukleinsäureamplifikationstest (NAAT) und/oder Kultur gelang in der Mehrheit der Fälle beider Kohorten (76 %) (47/61 Fälle [77 %] in Kohorte 1 und 15/21 Fälle [71 %] in Kohorte 2) (Tabelle 2). Der am häufigsten nachgewiesene Erreger war in beiden Kohorten *M. marinum* (87,2% in Kohorte 1, 66,7% in Kohorte 2), gefolgt von *M. abscessus/chelonae* (n = 5; davon n = 3 *M. chelonae* in Kohorte 1 und n = 1 in Kohorte 2 sowie n = 1 *M. abscessus*‐Komplex in Kohorte 2, weitere Speziesdifferenzierung nicht möglich). Weitere nachgewiesene Erreger waren *M. avium* und *M. haemophilum* (jeweils ein Fall in Kohorte 1) sowie *M. gilvum*, *M. vaccae* und *M. goodii* (jeweils ein Fall in Kohorte 2) (Tabelle 2).

**TABELLE 2:** Diagnostische Ergebnisse und therapeutische Strategien.

			Deutschland (2000–2011)	Köln (2012–2024)	Kombiniert (2000–2024)
Durchgeführte Diagnostik – n (%)	Histologie + NAAT + Kultur Kultur + Histologie Kultur + NAAT Histologie + NAAT Nur NAAT Nur Kultur Nur Histologie Nur klinische Symptome		21 (28,8) 19 (26,0) 9 (12,3) 7 (9,6) 1 (1,4) 4 (5,5) 10 (13,7) 2 (2,7)	21 (100) 0 (0) 0 (0) 0 (0) 0 (0) 0 (0) 0 (0) 0 (0)	42 (44,7) 20 (21,3) 9 (9,6) 7 (7,4) 1 (1,1) 4 (4,3) 9 (9,6) 2 (2,1)
Erregernachweis mit NAAT und/oder Kultur positiv – n (% der Fälle, in denen entweder NAAT oder Kultur oder beides durchgeführt wurde)	Ja Nein	Nur NAAT positiv Nur Kultur positiv Beide positiv	47 (77,0) 8 (12,9) 31 (50,8) 8 (12,1) 14 (23,0)	15 (71,4) 6 (28,6) 3 (14,3) 6 (28,6) 6 (28,6)	63 (75,9) 14 (16,9) 35 (42,2) 14 (16,9) 18 (21,7)
Detektierte Erreger – n (%)	*M. marinum* *M. chelonae* *M. abscessus* complex^*^ *M. avium* *M. haemophilum* *M. gilvum* *M. vaccae* *M. goodii* Nicht spezifiziert		41 (87,2) 3 (6,3) 0 (0) 1 (2,1) 1 (2,1) 0 (0) 0 (0) 0 (0) 1 (2,1)	10 (66,7) 1 (6,7) 1 (6,7) 0 (0) 0 (0) 1 (6,7) 1 (6,7) 1 (6,7) 0 (0)	52 (82,5) 4 (6,3) 1 (1,6) 1 (1,6) 1 (1,6) 1 (1,6) 1 (1,6) 1 (1,6) 1 (1,6)
Granulome in der Histologie – n (%)	Ja Nein		50 (87,7) 7 (12,3)	17 (81,0) 4 (19,0)	67 (85,9) 11 (14,1)
Zeit bis zur Diagnose (Tage)	Mittelwert (Bereich) Median (IQR)		199 3–2060 90 53–187	276,2 70–1108 151 118–315	214 3–2060 98 57–199
Periode zwischen vermuteter und bestätigter Diagnose in Fällen mit positivem Erregernachweis	Mittelwert (Bereich) Median (IQR)		n/a n/a	37,2 9–90 31 13,5–51,5	n/a n/a
Therapeutische Strategie (first‐line)	Nur antibiotische Therapie Antibiotische plus chirurgische Therapie Nur chirurgische Therapie Keine spezifische Therapie		62 (84,9) 7 (9,6) 2 (2,7) 2 (2,7)	19 (90,5) 0 (0) 0 (0) 2 (9,5)	81 (86,2) 7 (7,4) 2 (2,1) 4 (4,3)
Erstlinien‐Antibiotika‐Therapie – n (% der Fälle, in denen eine antibiotische Therapie initiiert war)	AB Monotherapie AB Kombinationstherapie (≥ 2 AB)	Kombination von 2 AB Kombination von 3 AB Kombination von 4 AB	47 (68,1) 22 (31,9) 13 (18,8) 8 (11,6) 1 (1,4)	10 (52,6) 9 (47,4) 6 (31,6) 2 (10,5) 1 (5,3)	57 (64,8) 31 (35,2) 19 (21,6) 10 (11,4) 2 (2,3)
Verwendete Erstlinien‐Antibiotika – n (% der Fälle, in denen eine antibiotische Therapie initiiert wurde)	Clarithromycin Doxycyclin Cotrimoxazol Clarithromycin + Rifampicin Minocyclin Rifampicin + Ethambutol Ciprofloxacin Doxycyclin + Cotrimoxazol Levofloxacin + Minocyclin Clarithromycin + Rifampicin + Ethambutol Clarithromycin + Doxycyclin Levofloxacin Clarithromycin + Rifampicin + Protionamid Clarithromycin + Rifampicin + Minocyclin Clarithromycin + Ethambutol Clarithromycin + Ciprofloxacin Clarithromycin + Levofloxacin Rifampicin + Ethambutol + Isoniazid + Pyrazinamid		19 (27,5) 13 (18,8) 10 (14,5) 5 (7,2) 5 (7,2) 4 (5,8) 2 (2,9) 2 (2,9) 2 (2,9) 2 (2,9) 0 (0) 1 (1,4) 1 (1,4) 1 (1,4) 1 (1,4) 1 (1,4) 0 (0) 0 (0)	6 (31,6) 2 (10,5) 1 (5,3) 3 (15,8) 1 (5,3) 0 (0) 0 (0) 0 (0) 0 (0) 2 (10,5) 2 (10,5) 0 (0) 0 (0) 0 (0) 0 (0) 0 (0) 1 (5,3) 1 (5,3)	25 (28,4) 15 (17,0) 11 (12,5) 8 (9,1) 6 (6,8) 4 (4,5) 2 (2,3) 2 (2,3) 2 (2,3) 4 (4,5) 2 (2,3) 1 (1,1) 1 (1,1) 1 (1,1) 1 (1,1) 1 (1,1) 1 (1,1) 1 (1,1)
Dauer der antibiotischen Therapie	Mean (Bereich) Median (IQR)		128 10–436 105 77–165	152,3 42–262 140 109,5–199,5	133 10–436 122 81–180
Komplikationen – n (%)	Ja Nein	Schmerz Superinfektion Verzögerte Wundheilung Kachexie	12 (16,4) 0 (0) 11 (15,1) 1 (1,4) 0 (0) 61 (83,6)	4 (19) 2 (10) 1 (5) 0 (0) 1 (5) 17 (81)	16 (17) 2 (2,1) 12 (12,8) 1 (1,1) 1 (1,1) 78 (83,0)
Nebenwirkungen der antibiotischen Therapie – n (%)	Ja Nein	Diarrhoe Toxische Dermatitis Tendinitis Hörverlust	1 (1,4) 0 (0) 0 (0) 1 (1,4) 0 (0) 72 (98,6)	5 (23,8) 3 (14,3) 1 (4,8) 0 (0) 1 (4,8) 16 (76,2)	6 (6,4) 3 (3,2) 1 (1,1) 1 (1,1) 1 (1,1) 88 (93,6)
Abschlussuntersuchung erfolgt – n. (%)	Ja Nein (*lost to follow‐up*)		13 (17,8) 60 (82,2)	15 (71,4) 6 (28,6)	28 (29,8) 66 (70,2)
Rezidiv – n (% der Fälle mit Abschlussuntersuchung)	Ja Nein		2 (15,4) 11 (84,6)	2 (13,3) 13 (86,7)	4 (14,3) 24 (85,7)

*Abk*.: AB, Antibiotikum/a; IQR, Interquartilsabstand; NAAT, Nukleinsäureamplifikationstest

* In diesem Fall war der NAAT positiv für *M. abscessus* complex, aber *M. abscessus* und *M. chelonae* waren nicht unterscheidbar.

Interessanterweise war bei den zwölf immunsupprimierten Patienten mit positivem Erregernachweis der Anteil anderer NTM als *M. marinum* signifikant erhöht (n = 5 Infektionen mit *M. abscessus/chelonae*, n = 1 Infektion mit *M. haemophilum*) im Vergleich zu immunkompetenten Patienten ohne weitere bekannte Risikofaktoren (zum Beispiel kosmetische Eingriffe) (p = 0,0004). In sechs Fällen mit Auslandsanamnese wurden folgende Erreger detektiert: *M. chelonae* (Thailand), *M. haemophilum* (Kenia, HIV‐positiver Patientin), *M. gilvum* (Ägypten) sowie *M. marinum* (Israel, Afghanistan und Slowakei jeweils ein Fall).

Antibiotika‐Resistenztestungen wurden in 24/40 Fällen (60%) aus Kohorte 1 und in 8/9 Fällen (89%) aus Kohorte 2 (alle in der Kultur *M.‐marinum*‐positiv) durchgeführt. Antibiotika‐Resistenzen wurden in 23/24 Fällen (96%) der Kohorte 1 und in 6/8 Fällen (75%) der Kohorte 2 festgestellt (Tabelle S1 im Online‐Supplement).

In 67/78 Fällen beider Kohorten (86%) zeigten sich histologisch Granulome. Bei den 17/21 (81%) Fällen mit Granulomnachweis aus Kohorte 2 war der häufigste Granulom‐Subtyp ein *messy granuloma* (8/19, 42%), gefolgt von nichtverkäsenden Granulomen in 6/19 Fällen (32%) und suppurativen Granulomen in 5/19 Fällen (26%). Eine Ziehl‐Neelsen‐Färbung auf säurefeste Bakterien wurde bei 14/21 (67%) Proben durchgeführt und war in allen Fällen negativ. Bei den vier Fällen ohne Granulombildung fanden sich histopathologisch entzündliche Infiltrate. Bei drei dieser vier Fälle wurde die Diagnose aufgrund eines positiven Erregernachweises gestellt und in einem Fall lediglich aufgrund des passenden Hautbefundes und anamnestischer Aquarium‐Exposition.

Die mittlere Symptomdauer bis zur Diagnosestellung betrug 199 Tage (Bereich: 3–260) in Kohorte 1 und 276 Tage (Bereich: 70–1108) in Kohorte 2 (Tabelle 2). In über 90% der Fälle war die Erstlinientherapie nach Diagnosestellung einer kutanen NTM‐Infektion eine orale Antibiotika‐Therapie (69/73 Fällen (94,5%) in Kohorte 1 und 19/21 Fällen (90,5%) in Kohorte 2), die in sieben Fällen (10%, alle in Kohorte 1) mit chirurgischen Eingriffen kombiniert wurde. Zwei Patienten der Kohorte 1 erhielten ausschließlich eine chirurgische Therapie, und vier Patienten (je zwei in Kohorte 1 und Kohorte 2) erhielten keine spezifische Therapie.

In der Mehrzahl der Fälle erfolgte eine initiale antibiotische Monotherapie (47/69 Fälle [68,1 %] in Kohorte 1, 10/19 Fälle [52,6 %] in Kohorte 2), am häufigsten mit Clarithromycin (19/69 Fälle in Kohorte 1, 6/19 Fälle in Kohorte 2), gefolgt von Doxycyclin beziehungsweise Cotrimoxazol (13 beziehungsweise 10 Fälle in Kohorte 1 und 2 bzw. 1 Fall in Kohorte 2) (Tabelle 2). Die häufigste Kombinationstherapie war Clarithromycin plus Rifampicin (5 Fälle in Kohorte 1 und 3 Fälle in Kohorte 2). Weitere eingesetzte Erstlinien‐Antibiotika sind in Tabelle 2 aufgeführt. In 6/21 Fällen der Kohorte 2 wurde die Therapie im Verlauf deeskaliert, meist auf Clarithromycin oder Doxycyclin mono (für Kohorte 1 lagen hierzu keine Daten vor). Interessanterweise wurden alle Patienten bis auf einer mit Antibiotika behandelt, gegen die *M. marinum* keine Resistenz zeigte. Ein Patient aus Kohorte 1 wurde mit Cotrimoxazol behandelt (bei Resistenz von *M. marinum*); er erhielt zusätzlich eine Abszessdrainage und der Befund heilte vollständig ab.

Die mittlere Therapiedauer bis zum Absetzen betrug 128 Tage (Bereich: 10–436) in Kohorte 1 und 152,5 Tage (Bereich: 42–262) in Kohorte 2 (Tabelle 2). Eine Abschlussuntersuchung wurde in Kohorte 2 häufiger dokumentiert (15/21 Fälle, 71%) als in Kohorte 1 (13/73 Fälle, 18%). Rezidive traten in beiden Gruppen bei jeweils zwei Fällen auf (Details in Tabelle S2 im Online‐Supplement).

## DISKUSSION

Aufgrund fehlender klinischer Studien stellen kutane NTM‐Infektionen weiterhin eine diagnostische und therapeutische Herausforderung dar. Bis heute wurden mehrere retrospektive Fallserien aus verschiedenen geografischen Regionen veröffentlicht. Nach unserem Kenntnisstand handelt es sich bei unserer Studie um die erste größere Fallserie kutaner NTM‐Infektionen in Deutschland. Wir konnten Daten von 94 Patienten aus zwei unabhängigen Kohorten über einen Zeitraum von 24 Jahren analysieren. In beiden Kohorten betrafen die meisten Fälle immunkompetente Männer. Als Haupterreger wurde *M. marinum* in über 80% der Fälle identifiziert, wobei eine Wasserexposition der häufigste Risikofaktor war. Darüber hinaus zeigt unsere Analyse eine lange mediane Zeit bis zur Diagnosestellung von 3 Monaten sowie eine mediane Therapiedauer von 4 Monaten, überwiegend mit einer antibiotischen Monotherapie. Es traten wenige Komplikationen oder Nebenwirkungen auf, die Rezidivraten waren gering.

Das Erregerspektrum unserer Untersuchung entspricht den Ergebnissen vergleichbarer Studien aus Europa und den USA, in denen ebenfalls *M. marinum* als häufigster Erreger identifiziert wurde.^11^, ^18^ Im Gegensatz dazu zeigen die meisten Studien aus asiatischen Ländern und Australien überwiegend RGM‐verursachte Infektionen.^12^, ^13^, ^26^ Auffällig ist, dass in diesen Studien chirurgische und kosmetische Eingriffe, die häufig mit RGM‐Infektionen assoziiert sind,^11^, ^13^, ^14^ der NTM‐Infektion deutlich häufiger vorangegangen waren. In unserer Fallserie waren RGM‐bedingte kutane NTM‐Infektionen selten und traten nur in einem Fall nach einem chirurgischen Eingriff (Liposuktion) auf. Zudem war diese Patientin mit Azathioprin immunsupprimiert. Alle fünf RGM‐ und die einzige durch *M.haemophilum*‐verursachte Infektion traten bei immunsupprimierten Patienten auf, was – trotz der insgesamt geringen Fallzahl von zwölf immunsupprimierten Patienten mit positivem Erregernachweis – auf einen Zusammenhang zwischen Immunsuppression und anderen NTM als *M. marinum* hindeuten könnte. Zwei dieser Infektionen erfolgten jedoch nach Auslandsreisen, was unterschiedliche Erregerexpositionen in den jeweiligen Ländern widerspiegeln könnte. Aufgrund des Kosmetik‐Tourismus^27^, ^28^ könnte auch in Deutschland zukünftig mit einem Anstieg RGM‐induzierter kutaner NTM‐Infektionen gerechnet werden.^11^, ^13^, ^14^ Prospektive Studien mit größeren Kohorten, die Infektionen unter Berücksichtigung von Risikofaktoren (Immunsuppression, Reisen, chirurgische/kosmetische Eingriffe, aquatische Exposition) analysieren, sind notwendig, um die Inzidenz genauer zu bestimmen. Wie in anderen Fallserien insbesondere für *M.marinum‐*Infektionen^18^, ^29^, ^30^ zeigt unsere Studie eine männliche Prädominanz. In anderen Studien mit vorwiegend RGM‐bedingten Infektionen nach chirurgischen Eingriffen dominieren hingegen weibliche Patientinnen.^11^, ^12^, ^14^ Ob dies auf geschlechtsspezifisch unterschiedliche Risikofaktoren (zum Beispiel häufigere kosmetische Eingriffe bei Frauen^27^, ^31^ beziehungsweise Aquarienhaltung als männlich dominiertes Hobby in Deutschland^32^) oder auf biologische Ursachen für die unterschiedliche Erregerverteilung zurückzuführen ist, bedarf weiterer Untersuchungen.

Ähnlich wie in anderen Studien^26^, ^33^ wurde die Diagnose kutaner NTM‐Infektionen in beiden Kohorten häufig erst mit einer Verzögerung von mehreren Monaten nach Symptombeginn gestellt und meist bei Vorstellung an akademischen Zentren überhaupt erst erwogen. Zur korrekten Diagnose war die Kombination klinisch‐pathologischer Befunde mit mikrobiologischen Analysen und dem Behandlungsverlauf notwendig, was mit anderen Studien übereinstimmt.^14^, ^30^ Die Standard‐Histopathologie inklusive säurefester Färbung weist bei NTM‐Infektionen eine geringe Sensitivität auf. Obwohl in den meisten Fällen, auch in unserer Studie, Granulome nachweisbar waren,^18^, ^29^, ^33^, ^34^ können diese auch durch andere Infektionen wie Tuberkulose, Leishmaniasis oder subkutane Pilzinfektionen verursacht werden. Die Nachweisrate säurefester Bakterien – 0% in unserer Studie – ist ebenfalls gering^18^, ^30^, ^34^ und nicht hilfreich für die Unterscheidung zwischen NTM und tuberkulösen Mykobakterien.^19^

Der direkte Erregernachweis stellt den Goldstandard in der Diagnostik von NTM‐Infektionen dar. Unsere Rate positiver Kulturen von 73,6% in Kohorte 1 und 42,8% in Kohorte 2 sind mit anderen Studien vergleichbar.^8^, ^30^ Die Nachweisraten hängen wesentlich von optimalen Kulturbedingungen ab, die zwischen *M. marinum* und anderen NTM differieren.^19^ Für bestätigte *M.marinum*‐Fälle betrug die Rate positiver Kulturen 92,1% (Kohorte 1) und 90% (Kohorte 2). Während positive Kulturen eine Resistenztestung und die gezielte Therapieauswahl ermöglichen, dauert die Kultivierung mehrere Wochen und erfordert spezielle Bedingungen.^19^ Der molekulare Nachweis mittels NAAT beziehungsweise Sequenzierung ist schneller und ermöglicht eine frühere Therapieeinleitung. In unserer Studie waren jedoch nur 42% (Kohorte 1) beziehungsweise 57% (Kohorte 2) der durchgeführten NAAT positiv. Interessanterweise wurden alle fünf NTM‐Infektionen außer *M. marinum* in Kohorte 2 ausschließlich molekular nachgewiesen, die Kulturen blieben negativ. Neuere Studien mit standardmäßiger NAAT‐Diagnostik berichten kaum über falsch‐negative Ergebnisse.^12^, ^14^, ^18^, ^26^ Ein negativer NAAT kann auf eine niedrige Mykobakteriendichte im Gewebe zurückzuführen sein. Andererseits bedeutet der Nachweis von NTM‐DNA nicht zwangsläufig eine Infektion, da Kontaminationen häufig sind.^19^ Die Differenzierung innerhalb des *M.abscessus/chelonae*‐Komplexes mittels NAAT kann herausfordernd sein,^35^ was problematisch ist, wenn keine Kultur für Speziesidentifikation und Resistenztestung vorliegt. Im Gegensatz zu *M. chelonae* kann *M. abscessus* das Erythromycin‐Resistance‐Methylase‐Gen (erm41) exprimieren, welches eine induzierbare Makrolidresistenz vermittelt ^36,37^ und die Auswahl des Antibiotikums beeinflusst. In unserer Studie betraf dies einen Fall (immunsupprimierte Patientin nach Liposuktion) (Abbildung 2), bei dem der NAAT *M. abscessus/chelonae*‐Komplex nachwies, eine weitere Differenzierung jedoch nicht möglich war. Nach sorgfältiger Abwägung und trotz des Risikos einer induzierten Makrolidresistenz wurde die Patientin 5 Monate oral mit Clarithromycin und Doxycyclin behandelt, was zu einer vollständigen Abheilung führte.

In Kohorte 2 erfolgte die Diagnostik standardisiert mit NAAT, Kultur und Histopathologie aus Biopsien, während in Kohorte 1 die diagnostischen Ansätze variabler waren. Dies erklärt sich durch das multizentrische Design von Kohorte 1 sowie den früheren Untersuchungszeitraum (NAAT damals noch nicht flächendeckend verfügbar). Aufgrund begrenzter Sensitivität und Spezifität von Kultur und NAAT sowie der Tatsache, dass manchmal nur eine der beiden Methoden einen Erreger nachwies, besteht die Notwendigkeit standardisierter Diagnostik‐Empfehlungen bei Verdacht auf NTM‐Infektionen: Vor empirischer antibiotischer Therapie sollten ausreichend große Hautbiopsien (zum Beispiel drei 6‐mm‐Stanzbiopsien) für Kultur, NAAT und Histopathologie entnommen werden. Kontaminationsquellen wie Leitungswasser sollten vermieden und vor Diagnostik kein Antibiotikum gegeben werden. Das mikrobiologische Labor ist auf die Verdachtsdiagnose hinzuweisen, um Proben optimal zu kultivieren (idealerweise parallele Kulturen bei 28–30 °C und 35–37 °C zur Abdeckung verschiedener NTM‐Spezies).^19^

In 15% der Fälle von Kohorte 1 wurde die Diagnose ausschließlich anhand klinisch‐pathologischer Befunde und dem Therapieansprechen gestellt, ein Vorgehen, das vor der Verbreitung von NAAT und Kultur üblich war.^30^ Es ist möglich, aber unwahrscheinlich, dass in diesen Fällen eine andere Diagnose vorlag. Die mediane Diagnosedauer von 3 Monaten entspricht anderen Studien^12^, ^13^, ^14^, ^26^, ^29^ und hing vom Diagnoseverfahren ab (NAAT und klinisch‐histopathologische Diagnosestellung schneller als Kultur). Da in Kohorte 2 die Diagnostik immer Biopsien für Kultur und NAAT einschloss, war die mediane Zeit bis zur Diagnose länger als in Kohorte 1, wobei die Diagnose in einigen Fällen ohne Erregernachweis gestellt wurde. Neben den Verzögerungen durch die komplexe Diagnostik wird damit deutlich, dass kutane NTM‐Infektionen oft erst nach Ausschluss anderer Diagnosen in Betracht gezogen werden. In unserer Studie wurden 20–30% der Fälle initial fehldiagnostiziert.

Die bevorzugte antimikrobielle Therapie und das Antibiotikaspektrum in unseren Kohorten entsprechen anderen Fallserien. Allerdings erhielten dort 50–80% der Patienten zusätzlich chirurgische Eingriffe.^11^, ^12^, ^14^, ^29^, ^33^, ^38^ Die Beobachtung, dass Antibiotikatherapie allein mit einer höheren Therapieversagensrate einhergeht,^7^ konnte in unserer Studie nicht bestätigt werden. Dies lässt sich möglicherweise durch den RGM‐Anteil und die Aufnahme tieferer oder invasiver Infektionen in anderen Studien erklären.

In den meisten aktuellen Fallserien wurde mit mindestens zwei antimikrobiellen Substanzen gleichzeitig behandelt,^12^, ^14^, ^29^, ^33^ andere berichten auch über gute Ergebnisse mit einer antibiotischen Monotherapie.^18^, ^32^, ^39^, ^40^ Das am häufigsten eingesetzte Antibiotikum in unserer Studie war Clarithromycin, gefolgt von Doxycyclin oder Cotrimoxazol. Die am häufigsten verordnete Kombination war Clarithromycin plus Rifampicin, entsprechend den Empfehlungen zur Behandlung pulmonaler *M.marinum*‐Infektionen von 2007.^20^ Verschiedene weitere Kombinationen und Monotherapien wurden in einzelnen oder wenigen Fällen angewandt.

Die Vielfalt der in dieser und anderen Studien eingesetzten Antibiotika unterstreicht die Notwendigkeit standardisierter Therapieschemata. Ein wesentlicher Grund hierfür ist das Fehlen evidenzbasierter Leitlinien, die auf kontrollierten Antibiotikastudien beruhen. Zudem stellen kutane NTM‐Infektionen eine seltene Gruppe von Hautinfektionen dar, die klinisch ähnlich erscheinen, jedoch durch unterschiedliche Erreger mit verschiedenen biologischen Eigenschaften (zum Beispiel Wachstumsgeschwindigkeit, optimale Inkubationstemperatur, Virulenzfaktoren, wirksame Antibiotika) verursacht werden, was eine Standardisierung zusätzlich erschwert. Ergebnisse zu *M.marinum‐*Infektionen – die in unserer Studie den Großteil der Fälle ausmachten – sind daher nicht ohne Weiteres auf andere NTM übertragbar.

Besonders hervorzuheben ist, dass bei einer geplanten empirischen Monotherapie mit Clarithromycin stets das Risiko einer makrolidresistenten M.*abscessus*‐Infektion bedacht werden sollte – insbesondere im Zusammenhang mit Infektionen nach operativen Eingriffen. Bei geringem Komplikationsrisiko empfiehlt es sich, die Behandlung bis zum Vorliegen der Speziesidentifikation und der Resistenztestung abzuwarten. Unsere mittlere Behandlungsdauer von 4 Monaten entspricht den Ergebnissen anderer Studien, welche Behandlungszeiten von 2,5 bis 6 Monaten beschreiben, mit tendenziell längeren Therapien bei durch RGM verursachten Infektionen.^12^, ^18^, ^26^, ^32^, ^33^

Das gute therapeutische Ergebnis mit niedrigen Rezidivraten in unserer Fallserie zeigt, dass eine (mono‐)antibiotische Therapie ohne chirurgischen Eingriff ausreichen kann, um kutane NTM‐Infektionen effizient zu behandeln. Klare Risikofaktoren für Rezidive konnten hauptsächlich aufgrund der geringen Fallzahl nicht identifiziert werden.

Unsere Studie hat mehrere Limitationen. Alle Daten wurden retrospektiv erhoben, und die beiden Kohorten unterschieden sich hinsichtlich Studiendesign (Kohorte 1 multizentrisch, Kohorte 2 monozentrisch) sowie Diagnose‐ und Behandlungszeitraum (Kohorte 1 2000–2012, Kohorte 2 2012–2024), was beim Vergleich der Daten zu berücksichtigen ist. Neben vielen Gemeinsamkeiten zeigen sich auch wesentliche Unterschiede (zum Beispiel längere Symptomdauer bis zur Erstvorstellung, längere Zeit bis zur korrekten Diagnose, längere Antibiotikatherapie und häufigere Nachsorge in Kohorte 2), die vermutlich zentrumsbedingt sind (standardisierte Anamnese, Diagnostik und Therapie in der monozentrischen Kohorte 2) und den medizinischen Fortschritt widerspiegeln. Die Interpretation der multizentrischen Daten aus Kohorte 1 ist aufgrund heterogener Dokumentation, Fallaufnahmen, diagnostischer Verfahren und Therapie weniger verlässlich. Eine detailliertere Analyse ist aufgrund fehlender Daten nicht möglich. Außerdem war die Anzahl von anderen NTM‐Spezies als *M. marinum* zu gering, um belastbare Aussagen zu treffen, insbesondere im Hinblick auf diagnostische und therapeutische Empfehlungen. Da ausschließlich Fälle aus dermatologischen Kliniken eingeschlossen wurden, sind Patienten aus anderen Fachabteilungen (zum Beispiel Infektiologie) oder mit tiefen NTM‐Infektionen, die meist chirurgisch behandelt werden, nicht berücksichtigt.

### Fazit

Die Diagnostik kutaner NTM‐Infektionen beruht auf der Kombination klinisch‐histopathologischer Befunde, mikrobiologischer Diagnostik und dem Therapieansprechen. Zur Steigerung der diagnostischen Ausbeute sollten vor jeder empirischen Antibiotikabehandlung ausreichend große Gewebeproben für mykobakterielle Kulturen, NAAT und Histopathologie entnommen werden. *Mycobacterium marinum* ist in Deutschland der häufigste Erreger kutaner NTM‐Infektionen. Es besteht ein Bedarf an standardisierten Therapieregimes. In unserer Kohorte war die orale Monotherapie mit Clarithromycin oder Doxycyclin über mindestens 4 Monate die meistverordnete Behandlung. Das gute Outcome bei niedrigen Rezidivraten spricht dafür, dass eine einzelne Behandlungsserie mit Clarithromycin oder Doxycyclin ausreichend sein kann, zumindest wenn keine *M.abscessus*‐Infektion vermutet wird.

## DANKSAGUNG

L.B. wurde durch das *Cologne Clinician Scientist Program* (CCSP), Fakultät für Medizin der Universität zu Köln, gefördert, welches von der *Deutschen Forschungsgemeinschaft* (FI 773/15‐1) finanziert wird, sowie durch das *Koeln Fortune Programm*/Fakultät für Medizin der Universität zu Köln. I.S. wird durch das DZIF (Fördernummern TI 07.001_SUAREZ_00, TI 07.001_SUAREZ_01 und TTU2.913_00) sowie den *Gemeinsamen Bundesausschuss* (G‐BA, Bundesministerium für Gesundheit) unterstützt. M.F. erhielt eine Förderung durch die *Deutsche Forschungsgemeinschaft* (FA849/5‐1). E.v.S. wurde von der *Deutschen Forschungsgemeinschaft* (Projekt‐Nr. 318346496, SFB1292/2 TP15) finanziell unterstützt. Die Förderer hatten keinen Einfluss auf Studiendesign, Datenerhebung und ‐auswertung, Publikationsentscheidung oder die Manuskripterstellung. Friedemann Reinhold wird für die Bildbearbeitung gedankt.

Open access Veröffentlichung ermöglicht und organisiert durch Projekt DEAL.

## INTERESSENKONFLIKT

Keiner.

## Supporting information



Supplementary information
