# Supplementary material for: Kutane Infektionen mit nicht‐tuberkulösen Mykobakterien: eine retrospektive Studie mit 94 Fällen aus Deutschland
Source: J Dtsch Dermatol Ges. 2026 May 5;24(5):608–18. [Article in German] doi: 10.1111/ddg.15910_g (PMC13140136; doi:10.1111/ddg.15910_g)
Supplement: Supplementary file 1 — Supplementary information [file DDG-24-608-s001.docx]

|  |  | Deutschland  (2000-2011) | Köln  (2012-2024) |
| --- | --- | --- | --- |
| **Resistogramm erfolgreich** – Nr. (% der Fälle mit positiver Kultur) | Ja  Nein | 24 (60)  16 (40) | 8 (88.9)  1 (11.1) |
| **Antibiotika-Resistenzen** – Nr. (% aller gefundenen Resistenzen) | Isoniazid  Pyrazinamid  Rifampicin  Ethambutol  Streptomycin  Trimethoprim-sulfamethoxazol  Levofloxacin  Ciprofloxacin  Moxifloxacin  Doxycyclin  Rifabutin | 21 (34.4)  18 (29.5)  0 (0)  0 (0)  13 (21.3)  1 (1.6)  4 (6.6)  3 (4.9)  0 (0)  0 (0)  0 (0) | 0 (0)  0 (0)  2 (13.3)  3 (20)  0 (0)  3 (20)  0 (0  4 (26.7)  1 (6.7)  1 (6.7)  1 (6.7) |

**Supplemental Table S1. Nachgewiesene Antibiotika-Resistenzen in beiden Gruppen.** Anmerkung: Je nach Zentrum wurden unterschiedliche Resistenz-Panel angewandt.

| **Patient (P-Nr., Kohorte) und Risikofaktor(en)** | **Diagnose mittels** | **Verschriebene Erstlinientherapie** | **Therapie nach dem Rezidiv und weiterer Verlauf** |
| --- | --- | --- | --- |
| P53, Kohorte 1 | Erregernachweis (multipel-resistenter *M. marinum*) | Rifampicin plus Ethambutol, unbekannte Dauer | Lost to follow-up nach Rezidiv-Diagnose |
| P62, Kohorte 1, Immunsuppression mit ciclosporin | Erregernachweis (multipel-resistenter *M. marinum*) | Doxycyclin über 135 Tage | Wechsel auf Rifampicin plus Clarithromycin plus Ethambutol über 14 days, gefolgt von Clarithromycin mono über 70 Tage, dann Ausheilung der Infektion |
| P08, Kohorte 2, Immunsuppression mit Etanercept | Kombination aus klinischen und histopathologischen Befunden | Clarithromycin, Ethambutol und Rifampicin über 4 Monate, Rezidiv nach 2 Jahren | Re-Initiierung der Triple-Therapie, weiter anlaufend |
| P11, Kohorte 2 | Erregernachweis (*M.marinum*) | Clarithromycin für 69 Tage, Rezidiv nach einem Monat | Re-Initiierung von Clarithromycin, dann lost to follow-up |

**Supplemental Table S2. Überblick der Fälle mit rezidivierenden NTM-Infektionen und mögliche Erklärung für das Rezidiv.**
